# Comprehensive genome and transcriptome analyses reveal genetic relationship, selection signature, and transcriptome landscape of small-sized Korean native Jeju horse

**DOI:** 10.1038/s41598-019-53102-8

**Published:** 2019-11-13

**Authors:** Krishnamoorthy Srikanth, Nam-Young Kim, WonCheoul Park, Jae-Min Kim, Kwon-Do Kim, Kyung-Tai Lee, Ju-Hwan Son, Han-Ha Chai, Jung-Woo Choi, Gul-Won Jang, Heebal Kim, Youn-Chul Ryu, Jin-Wu Nam, Jong-Eun Park, Jun-Mo Kim, Dajeong Lim

**Affiliations:** 1 Animal Genomics and Bioinformatics Division, National Institute of Animal Science, Rural Development Administration, Wanju, 55365 Republic of Korea; 2 Subtropical Livestock Research Institute, National Institute of Animal Science, Rural Development Administration, Jeju-do, 63242 Republic of Korea; 3 Cancer Genetics and Comparative Genomics Branch, National Human Genome Research Institute, National Institutes of Health, Bethesda, MD 20892 USA; 4 C&K genomics, Seoul, Republic of Korea; 5 Animal Breeding and Genetics Division, National Institute of Animal Science, Rural Development Administration, Wanju, 55365 Republic of Korea; 6 College of Animal Life Science, Kangwon National University, Chuncheon, 24341 Republic of Korea; 7 Division of Biotechnology, Jeju National University, Jeju, 63243 Republic of Korea; 8 Department of Life Science, Hanyang University, Seoul, 133-791 Republic of Korea; 9 Department of Animal Science and Technology, College of Biotechnology and Natural Resources, Chung-Ang University, Ansung-si, 17546 Republic of Korea

**Keywords:** Agricultural genetics, Structural variation, Comparative genomics

## Abstract

The Jeju horse, indigenous to the Jeju Island in Korea may have originated from Mongolian horses. Adaptations to the local harsh environment have conferred Jeju horse with unique traits such as small-sized body, stocky head, and shorter limbs. These characteristics have not been studied previously at the genomic level. Therefore, we sequenced and compared the genome of 41 horses belonging to 6 breeds. We identified numerous breed-specific non-synonymous SNPs and loss-of-function mutants. Demographic and admixture analyses showed that, though Jeju horse is genetically the closest to the Mongolian breeds, its genetic ancestry is independent of that of the Mongolian breeds. Genome wide selection signature analysis revealed that genes such as *LCORL*, *MSTN*, *HMGA2*, *ZFAT*, *LASP1*, *PDK4*, and *ACTN2*, were positively selected in the Jeju horse. RNAseq analysis showed that several of these genes were also differentially expressed in Jeju horse compared to Thoroughbred horse. Comparative muscle fiber analysis showed that, the type I muscle fibre content was substantially higher in Jeju horse compared to Thoroughbred horse. Our results provide insights about the selection of complex phenotypic traits in the small-sized Jeju horse and the novel SNPs identified will aid in designing high-density SNP chip for studying other native horse breeds.

## Introduction

Jeju is the largest island in the Korean peninsula^1^ and has a climate that is distinct from that of the Korean mainland. The Jeju island is home to several indigenous livestock species, such as chickens, pigs, dogs, and horses, which have been bred as closed flock^2^. Among the indigenous species, the Jeju horse (JH) is one of the most important animals from an historical, cultural, and economical perspective^3^. Horses were domesticated in the Eurasian steppe over 6,000 years ago^4^ and have had a lasting impact on human civilization and lifestyle by influencing mobility, trade, food, and warfare^5^. The JH is a landrace with extensive history; it is the only Korean native horse^3^ and is registered with the Domestic Animal Diversity Information System of the Food and Agriculture Organization (FAO)^6^. Starting from the early 1970s, the population of the JH declined rapidly and was close to extinction by early 1980s^3^. This occurred because the demand for horses declined owing to industrialization and development of modern means of transportation and agricultural machinery^7^. Realizing the historical and cultural importance of JH and the need for its conservation, the Korean government designated the JH as a Korean National Treasure (No. 347) in 1986, and started a pedigree registry^3,7^. In 2000, a JH registration agency was established, and currently, approximately 2,500 JH are being raised at local farms^7,8^.

Several studies have examined the origin of the JH based on historical^9^, archaeological^10^, and molecular evidence^11–14^; however, the origin of these horses remains unclear. Based on historical records, Nam (1969) suggested that in 1276 AD, 160 horses were introduced to the Jeju Island by the Mongolian Yuan dynasty of China, and that these horses were bred for their warfare capabilities^9^. However, based on archaeological evidence^10,12^, horses seem to have inhabited the Jeju Island 2500 years prior to the Mongolian invasion. Moreover, the results of several studies have suggested multiple origins for JH^11,13–15^. Based on restricted fragment length polymorphism (RFLP) analysis of mtDNA, Oh *et al*. suggested in 1994 that JH are closer to Mongolian wild horses (*E. przewalskii*)^12^. However, the genetic origins of the JH are still incompletely understood, because these studies used a limited number of samples and did not conduct whole genome comparative analyses. In this study, we performed a whole genome comparative analysis of the JH and 5 other breeds, including Thoroughbred horses (TB), 3 Mongolian breeds (MH) (Mongolian Galshar (MG), Mongolian Domestic Horse (MD), Mongolian Jarglant (MJ)) and Przewalski’s (PZ) horses. The JH, which is found only in the Jeju island of Korea, is a small-sized hardy horse (Supplemental Figure S1). On average, it has a wither height of ~122 cm, which is shorter than those of the average MH (~140 cm), PZ (~148 cm), and TB horses (~154 cm)^6,7,11,16–18^. JH are gentle, hardy, and of a healthy constitution, and are resistant to diseases and stress derived from the harsh environment they live in^3^. The JH shows remarkable endurance and stamina and are raised for meat, farm labour, and riding^19^, however they are not intensively selected for any one purpose^20,21^. While TB have been intensively selected for speed, stamina, and agility^22^, PZ is the only species of wild horse surviving in the world today and is native to Mongolia^23,24^. The combined effect of environmental changes and human activities has drastically reduced the PZ population, with only 12 individuals surviving in the middle of the last century^24–27^. Currently, the number of PZ has increased to approximately 1,000–2,000; these horses are found in the wild and in zoos across the world^24,26^. In comparison, the population of MH, which includes breeds such as Galshar, Darkhad, Tes, Myangad, and Jargalant^28^, is large, with abundant genetic diversity, as they have been an integral part of nomadic pastoralist culture in North Asia. Comparative genomic analysis of these breeds can provide insights about the genetic origin of the JH, and can confirm whether the JH originated from a MH breed. Natural selection, domestication, and adaptation to local environments can cause phenotypic changes via mutations^29^, while robust selection of beneficial alleles leads to selective sweep^29^. These genomic footprints, which are left by selection, are known as selection signatures, and can be used to identify loci that were subjected to selection. Although JH was not subjected to artificial selection, the genetic consequences of naturally occurring positive selection are essentially the same as those of artificial selection^30–33^. Selective sweep analysis can identify loci that were selected in response to local adaptations. Similarly, comparative transcriptome analysis of JH and TB horses can provide molecular clues and help identify genes that influence traits such as stamina, endurance, hardiness, and small wither height and the poor athletic ability observed in JH.

The aims of this study were to: 1) examine the genetic relationship of the JH and Mongolian horses; 2) understand the demographic history of the JH; and 3) identify the genetic basis of selective and adaptive functional differences between the JH and TB. For this, we conducted whole-genome re-sequencing analysis of 41 horses belonging to 6 breeds including JH, TB, and 3 Mongolian horse breeds (Galshar, Jarglant, and Mongolian domestic horse), and performed RNA sequencing-based comparative transcriptome analysis between TB and JH using the lung, rump muscle, thigh muscle, liver, and heart tissues. We believe the results of this study will provide cues towards the genetic origin of the JH and also identify candidate genes that play a role in the small size and poor athletic performance of JH.

## Results and Discussion

### Resequencing, and SNP detection in 41 s horses

A total of 16.5 billion initial reads were generated by the Illumina HiSeq 4000 Sequencer, amounting to an average of 3.8 Gbp per library (average of 16.37X fold coverage across the genome, individual fold coverage ranged between 12.26X~23.28X) (Table 1, Supplementary File 1). The highest average fold coverage was obtained for PZ (20.72X), while the lowest average fold coverage was obtained for MG (13.9X). Potential polymerase chain reaction duplications were removed from the aligned reads, and the reads were further modified using Picard toolkit before SNP calling. Approximately, 16.15 billion reads, with an average alignment rate of 97.15%, were mapped against the reference genome, with the highest average alignment rate for TB (98.72%), and the lowest for MJ (96.23%).

**Table 1:** Summary of sequencing, mapping, and coverage details.

Breed of horse	No of Sample	Raw_reads	Mapped_reads	Paired_reads	Alignment Rate^†^	Avg_fold^‡^
Mongolian Galshar	6	2056771059	2002716326	1693557220	96.45%	13.9 X
Mongolian Domestic Horse	4	1492500192	1453274027	1220426606	96.35%	15.16 X
Mongolian Jarglant	5	1798034653	1754687974	1481970526	96.23%	14.64 X
Jeju Island	12	4716082872	4630806096	3708745506	97.97%	16.1 X
Thoroughbred	10	4326958817	4248380754	3562897400	98.72%	17.7 X
Przewalski’s	4	2128278554	2063427021	1897781050	97.18%	20.72 X
Total	41	16518626147	16153292198	13565378308	97.15%	16.37 X

^†^Alignment rate is the percentage of reads that were aligned with the reference genome by the bowtie aligner.

^‡^Avg_fold– Average fold is the average coverage depth across the genome.

**Figure 1:**
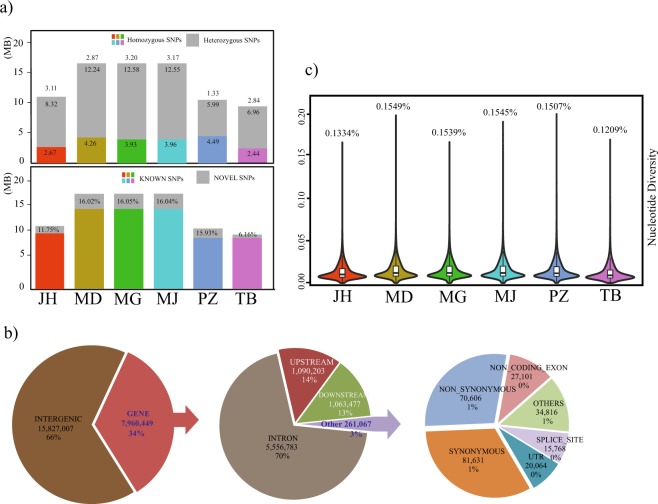
Variant statistics, SNP annotation, and missense and loss of function (LOF) mutants detected in this study. (**a**) Top panel shows the number of homozygous and heterozygous SNPs (per million base-pairs) identified in this study; the number on top of the bar indicates the ratio of heterozygous to homozygous SNPs. Bottom panel shows the number of known and novel SNPs identified (per million base-pairs), with the percentage of novel SNPs provided at the top of the bars. (**b**) Nucleotide diversity ratios across the genome of the six breeds analyzed in this study. (**c**) Venn diagrams showing the total number of MS, fixed MS, LOF, and fixed LOF mutants in the JH and TB.

Altogether, across the genome of the different breeds, we identified a total of 21,651,273 SNPs (Table 2, Supplemental File S1). The number of SNPs in each breed varied between 9.4 million (TB) and 16.5 million (MD, MJ and MG) (Table 2 and Fig. 1a). Additionally, 5,185,635 (23.95%) of the identified SNPs were novel, when compared against the dbSNP database build 151; this was expected, because the reference genome (EquCab 2.0) was based on a female TB horse^34^. Amongst the identified SNPs, the highest percentage of novel SNPs was in the Mongolian breeds (~16.07%), while the lowest percentage of novel SNPs was in TB (6.16%) (Fig. 1a); 15.93% and 11.75% of SNPs identified in PZ and JH were novel (Fig. 1a). However, there was considerable within-population variation in MH; this was reflected by the higher mean autosomal nucleotide diversity among the Mongolian breeds (0.1549%, 0.1539%, and 0.1507% for MD, MG, and MJ, respectively) (Fig. 1b) when compared with those of PZ (0.1507%), JH (0.1334%), and TB (0.1209%). Nucleotide diversity is the per site average nucleotide difference between any two randomly chosen DNA sequences within a population^35^. The reduced nucleotide diversity in TB is likely due to generations of intensive artificial selection^36^. JH may have undergone a certain level of inbreeding owing to its long-term isolation and small population size^6^. Moreover, the population of JH was drastically reduced in the 1970s^3^. The low nucleotide diversity indicates that carefully planned breeding and conservation strategies are needed to maintain the genetic diversity of the JH. Presently, all the PZ horses trace their origin to 12 members of the founding population^24–27^; however, PZ horses show relatively high mean nucleotide diversity, similar to that reported by Goto *et al*.^25^. This may be due to the carryover of pre-existing elevated genetic diversity in the PZ horses, which occurred prior to the genetic bottleneck of the last century and before subsequent interbreeding of the PZ horses with domestic horses^25,27^. The quality of our SNP data was analyzed by calculating the transition to transversion ratio (Ts/Tv) (Table 2), which is used as an indicator of sequencing and SNP data quality in cattle, humans, pigs, and horses^29,37–40^. We recovered a Ts/Tv ratio of 1.97 across the genome, which is similar to the global Ts/Tv ratio of 1.92–2.2, reported in previous studies^29,37–39^.

**Table 2:** Summary of all the SNPs identified in this study.

Categories	Total	JH	TB	PZ	MD	MG	MJ
Samples	41	12	10	4	4	6	5
SNPs	21651273	10993471	9410065	10482878	16513676	16521183	16516855
Transition	14371118	7280776	6211755	6961756	10998372	11001791	10999640
Transversion	7280155	3712695	3198310	3521122	5515304	5519392	5517215
Ts/Tv	1.97	1.95	1.91	1.95	1.99	1.99	1.99
**SNP categories** ^**†**^							
Synonymous coding	85436	43495	37033	41332	78412	78428	78419
Non-synonymous coding	73582	36033	30823	34080	53362	53413	53358
Start lost	85	35	35	37	68	69	69
Stop gained	868	371	325	346	623	626	623
Stop lost	48	35	14	37	38	38	38
Non-coding exon	27928	14596	12294	11570	19196	19235	19239
5′ Untranslated Region	6732	3685	3186	3551	6845	6853	6852
3′ Untranslated Region	12868	6042	5110	5964	10953	10955	10954
Splice site acceptor	561	358	315	335	491	495	496
Splice site donor	792	476	445	467	713	714	714
Intron	5714650	2844468	2421133	2756982	5484660	5485747	5484270
Intergenic	15827007	8094428	6942116	7676009	12077339	12083932	12080078
**Functional categories**^**‡**^							
missense	73562	36099	30872	34134	53362	53413	53358
LOF	1483	783	694	725	1150	1156	1154

^†^SNPs categorized based on their effects,. ^‡^Coding SNPs categorized based on type of mutation.

### SNP annotation and function assessment of missense and loss-of-function SNPs

SNPs were functionally annotated using SnpEff^41^ (Fig. 1c and Table 2). Most of the SNPs (66%) were located in the intergenic regions. Among genic SNPs, intronic SNPs (70%) were the most common, followed by SNPs located upstream (14%) and downstream (13%) of the genes. Only 3% of the genic SNPs were located in the exonic region. We identified a large number (73,562) of missense mutations (Table 2), which were part of 15,427 genes; some of this mutation may be associated with traits of interest (Supplemental File S2). We also identified 36,033, 30,872, 53,362, 53,413, 53,358 and 34,1334 missense mutations, located in 11,764, 10,948, 13,825, 13,829, 13,822 and 11,613 genes in the JH, TB, MD, MG, MJ and PZ populations, respectively (Table 2 and Supplemental File S2). Among the identified missense mutations, we found 4,108 (in 2,733 genes), 2,459 (in 1,512 genes), 3,635 (in 2,362 genes), 3,749 (in 2,465 genes), 4,181 (in 2,643 genes) and 4,915 (in 3,085 genes) SNPs to be segregating (i.e. fixed) in JH, TB, MG, MJ, MD and PZ respectively, compared to the reference genome (Supplemental File S3). Comparative analysis of the missense mutations between JH and TB revealed that 21,661 missense SNPs, located in 8,988 genes, were found in both the breeds, however, 14,438 missense SNPs, located in 7,430 genes, and 9,211 missense SNPs, located in 5,525 genes, were unique to JH and TB respectively (Supplemental File S4). Among the fixed missense mutations, 2,105 (found in 1,655 genes) and 2,003 (found in 1,288 genes) SNPs were found only in JH and TB respectively, while 456 fixed missense SNPs (found in 359 genes) were found in both JH and TB (Supplemental File S4). Gene ontology (GO) and KEGG pathways analyses showed that in JH (Table 3), genes with fixed missense mutations were enriched in negative regulation of the Wnt signalling pathway, central nervous system development, metallopeptidase activity, ATPase activity, ECM receptor interactions, and peptide catabolic process. In TB, genes with missense mutations were enriched in cerebellar development, positive regulation of dendritic spine development, binding of protein kinase A, and negative regulation of canonical Wnt signalling pathway.

**Table 3:** Gene Ontology analysis of missense and loss of function mutants fixed in JH and TB populations.

Breed	Type	Category	Term	Count	Genes
**JH**	**Missense mutations**	BP	Peptide catabolic process	7	ACE TRHDE TPP1 ANPEP LTA4H ENPEP RNPEP
		BP	Negative regulation of canonical Wnt signalling pathway	16	CTNND1 NOTUM WWTR1 PTPRO LATS2 ZNRF3 DDIT3 GLI1 GPC3 SFRP2 DACT3 SFRP4 LIMD1 TMEM88 APC AXIN1
		BP	Central nervous system development	9	NRCAM HAPLN2 B3GNT5 TPP1 ACAN CASZ1 BCAN ADGRA2 NCAN
		MF	Metalloendopeptidase activity	21	ADAM28 ADAMTS17 ADAM11 MMEL1 THOP1 ADAMTS16 MMP16 PAPLN MMP15 ADAM33 ADAM17 ADAM18 ATP23 ADAM19 ADAMTS12 PMPCA ADAMDEC1 PMPCB OMA1 ADAM9 ADAMTS4
		MF	ATPase activity	19	KIF14 KIFC1 DNAH10 KIF24 MLH1 MYO9B DNAH7 DNAH8 RNF213 DNAH6 CARNS1 TOR3A MACF1 VWA8 ATP5I MDN1 KIF21B PMS1 ABCA12
		KEGG	ECM-receptor interaction	15	COL4A4 ITGB4 ITGA9 LAMB4 SDC1 LAMA4 COL6A6 LAMA5 LAMC3 COMP TNR COL6A3 TNN RELN ITGA2B
		KEGG	Dilated cardiomyopathy	13	ITGA9 ADCY4 ADCY7 MYL2 TNNC1 ADCY5 ADCY6 ITGB4 RYR2 CACNA1F CACNA2D3 CACNA2D2 ITGA2B
	**LOF mutations**	BP	Apoptotic process	7	CLPTM1L PRUNE2 CYCS BIRC6 SIAH2 BRAT1 CIDEC
		MF	Glucose transmembrane transporter activity	2	SLC2A9 SLC2A10
		CC	Voltage-gated calcium channel complex	2	CACNA2D2 CACNA2D4
		KEGG	Adrenergic signalling in cardiomyocytes	3	ADCY5 RAPGEF4 CACNA2D2
		KEGG	Retrograde endocannabinoid signalling	8	SLC32A1 PLCB3 GABRA3 GABRA6 ADCY5 GRM1 PLCB2 CACNA1B
		BP	Intracellular signal transduction	14	PLCB3 PDZD8 MAST1 MAST2 NUAK2 SNRK RPS6KA2 SPSB3 ADCY5 DEF8 PLCD3 ASB16 PLCB2 DVL1
**TB**	**Missense mutations**	BP	Positive regulation of dendritic spine development	3	CAMK1 SHANK3 NEURL1
		BP	Cerebellum development	3	PTF1A GBX2 RPGRIP1L
		BP	Negative regulation of canonical Wnt signalling pathway	6	HDAC1 DACT3 SOX2 RGS19 SIAH2 SOX9
		MF	Protein kinase A binding	3	SPHKAP DACT2 DACT3
	**LOF mutations**	BP	Regulation of establishment or maintenance of cell polarity	2	LLGL1 LLGL2
		BP	Intraciliary retrograde transport	2	TTC21B TTC21A
		BP	Regulation of Notch signalling pathway	2	LLGL1 LLGL2
		KEGG	Calcium signalling pathway	6	ATP2B1 ADCY7 PHKB HRH2 CACNA1I PDGFRB
		KEGG	MAPK signalling pathway	6	TAOK2 CACNA1I PDGFRB FAS DUSP8 FLNA

BP – Biological Processes, MF – Molecular Function, CC – Cellular Components, KEGG – Kyoto Encyclopedia of genes and genomes.

To identify SNPs underlying recessive traits, we grouped protein coding SNPs belonging to five high-impact severity types; including SNPs functionally classified as start loss, stop gain, splice acceptor, splice donor and stop loss, into loss of function (LOF) variants. In total, we detected 1,483 LOF SNPs (LOFs) across the populations (Table 2 and Supplemental File S5), with the MH breeds carrying the greatest number of LOF SNPs (MG -1,156, MJ - 1,154 and MD - 1,150). This may be due to larger number of variants identified in the MH breeds relative to the other breeds analyzed in this study. In the JH (824) and TB (737), 287 LOFs were unique to the JH (Table 4) and 198 LOF SNPs were unique to TB, while 496 LOF SNPs were found in both the JH and TB (Supplemental File 7), Out of this we identified 43 and 34 LOF (LOF) SNPs to be fixed only in JH and TB populations, while 107 fixed LOF were found in both JH and TB (Table 4, Supplemental File 7). Comparative analyses of LOF (LOF) variants among the breeds may uncover genes that, although not necessary for survival and reproduction, may have acted as a means for adaptive evolution against environmental changes, thereby contributing to the phenotypic differences between breeds^42,43^. There were 140 and 130 genes with LOF variants in JH and TB populations, respectively (Supplemental File S6). Our GO and KEGG pathway analyses of genes affected by LOF revealed that genes involved in voltage gated calcium channel complex, glucose transmembrane transporter activity, intracellular signal transduction, and apoptosis, were overrepresented in JH; in TB, the genes affected by LOF were enriched in the MAPK signalling pathway, calcium signalling pathway, and regulation of Notch signalling pathway (Table 4). Though determining the effect of each one of the identified MS and LOF variants on gene expression was beyond the scope of this study, several promising candidate SNPs, previously found to be associated with critical traits of interest, were identified in the JH (Table 5).

**Table 4:** List of missense and loss of function mutation in jeju and thoroughbred horse

Type of Mutation	Unique to JH	Unique to TB	Shared
Fixed missense	2105	2003	4565
Total missense	14438	9211	21661
Fixed LOF	43	34	107
Total LOF	287	198	496

LOF – loss of function.

**Table 5:** Genetic variants associated with conformation and athletic performance traits in the Jeju horse.

Gene	CHR	Position (bp)	Associated Phenotype	Associated Genotype	Jeju Horse Genotype	Genotype Frequency	SNP_ID	Selection Signature (significance)
SCL26A2^107^	14	27301621	Autosomal recessively inherited chondrodysplasia	A > G	GG	1	rs393849014	
LCORL/NCAPG^58^	3	107374136	Body size	C > T	TT	1	rs68603064	ZHp (−3.6)
LASP1^58^	11	23334511	Body size	G > A	GG, GA, AA	0.25/0.5/0.25	rs68876319	
HMGA2^56^	6	82644800	Body size	C > T	TT	1	rs68671073	XPEHH (0.0034)
PROP1^71^	14	3000031	Dwarfism	G > C	GG/GC/CC	0.17/0.42/0.42	rs68963991	ZHp (−3.9)
PROP1^71^	14	3000132	Dwarfism	T > C	TT/TC/CC	0.33/0.5/0.17	rs68963993	ZHp (−3.9)
ACTN3^75^	12	30237132	Muscle fibre composition	T > G	TT	1	rs1146699694	XPEHH (0.004)
ACTN3^74^	12	30226577	Muscle fibre composition	G > A	GA, AA	0.42/0.68	rs1144978872	XPEHH (0.004)
MSTN^82^	18	66608679	Racing distance/ muscle fibre composition	C > T	TT, CT	0.9/0.1	rs397152648	ZHp(−3.9)
ACTN2^50^	1	75822632	Athletic performance	A > G	AA	1	rs68450030	XPEHH(0.005)
COX4I1^50^	3	33460779	Athletic performance	C > T	CC, CT	0.5/0.5	rs68518550	XPEHH(0.005)
COX4I2^49^	22	23314524	Athletic performance	C > T	CC	1	rs69276449	XPEHH(0.002)
ACN9^50^	4	40343817	Athletic performance	C > T	TT	1	rs69505998	
CKM^49^	10	16079732	Athletic performance	G > A	GG, GA	0.8/0.2	rs68819557	XPEHH(0.002)
PDK4^18^	4	39020227	Athletic performance	C > A	CC	1	rs69586787	XPEHH(0.003)
PDK4^50^	4	39024151	Athletic performance	G > A	GG	1	rs69586789	XPEHH(0.003)
DMRT3^81^	23	22391254	Pattern of locomotion	C > A	CC, CA, AA	0.25/0.5/0.25	rs1150690013	XPEHH(0.004)
PON1^50^	4	38747736	Athletic performance	C > T	CC, CT, TT	0.67/0.25/0.08	rs69585308	
ZFAT^55^	9	76901355	Wither height	C > T	TT	1	rs68748127	XPEHH(0.005)
ZFAT^56^	9	77656570	Wither height	C > T	CC	1	rs68750453	XPEHH(0.005)
ZFAT^55^	9	76901431	Wither height	C > A	AA	1	rs68748129	XPEHH(0.005)
ZFAT^55^	9	76901578	Wither height	G > A	AA	1	rs68748130	XPEHH(0.005)
ZFAT^55^	9	76904485	Wither height	G > A	AA, GA	0.92/0.08	rs68748134	XPEHH(0.005)

### Population structure, admixture proportions, demography, and migration events

**Figure 2:**
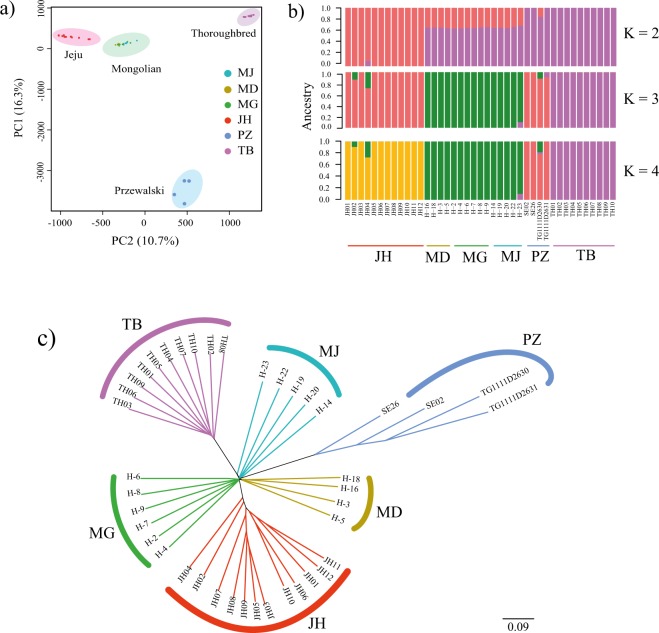
Results of population genomic analysis. (**a**) PCA plot of populations of the six horse breeds; principal components 1 and 2 indicate 16.3 and 10.7% of the observed variance, respectively. (**b**) Population structure of the 41 horses in the six breeds; the length of each colored segment represents the proportion of the genome inferred from the ancestral population (K). (**c**) Neighbour joining tree inferred from identity by descent distance matrix. The clades are colored according to breeds.

We used principal component analysis (PCA) and admixture analysis, based on genotypic data, to examine the genetic relationship among the sequenced horse populations. For PCA, we used only autosomal SNPs, and analysis was conducted using GCTA (version 1.25). The first and second principal components accounted for 16.3 and 10.7% of total variation (Fig. 2a). The individuals from the JH, MH (MJ, MG, and MD), TB, and PZ populations were grouped according to their origins using PCA (Fig. 2a). Our results indicated that the JH and MH were genetically closer, while TB and PZ horses were genetically distinct to the other breeds in the study. To estimate individual ancestry, admixture proportions were assessed without defined population information using ADMIXTURE (version 1.3.0)^44^ (Fig. 2b). The best K (number of ancestral population) was identified based on five-fold cross validation of the data. The individual population was grouped into separate clusters at K = 3 with the lowest cross validation error. The grouping of the JH into an independent unique cluster indicated that the ancestry of the JH was independent of that of MH, even though two individuals, JH 02 and JH 04, among the JH population showed 10 and 25% admixture with Mongolian genome, and this did not change even when K was increased to 4. This result indicates that within the JH population, some JH horses have Mongolian admixture across their genome, possibly because of the cross breeding of several native JH with MH in the past. This was reflected in the results of neighbour-joining (NJ) tree analysis, in which the two individuals (JH 02 and JH 04) were placed outside the main cluster (Fig. 2c).

**Figure 3:**
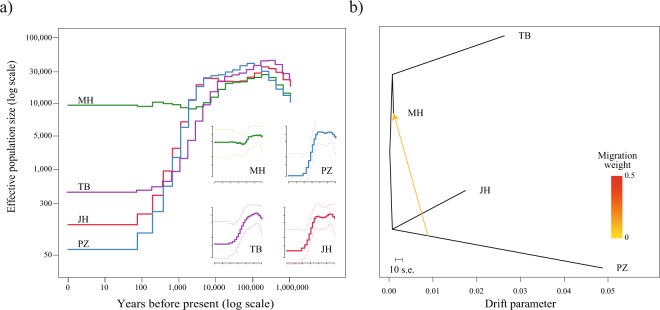
Historical demography and gene flow analysis. (**a**) Estimated effective population size of MG, TB, JH, and PZ populations. Inset shows the 95% confidence interval for the estimates. (**b**) Pattern of population splits and gene flow between the breeds. The drift parameter is proportional to effective population size (*Ne*). The migration edge is coloured according to the percentage of ancestry received from the donor. Scale bar shows the average standard error (10 times) of the entry in the sample co-variance matrix.

We then estimated past effective population size (Ne) of each breed in order to understand the demographic history of each breed (Fig. 3a). Considering their evolutionary history of extinction in the wild before successful captive breeding^24–27^, PZ experienced the greatest decline in population when compared with populations of other horse breeds examined in this study. The demographic history of the JH resembles that of PZ horses, which is compatible with the recent severe population reduction of JH^3^. MH was distinct from the other breeds, showing less fluctuation in population size over time, which reflects the abundant genetic diversity of MH^26^. Considering that the actual population of JH is closer to 2500 and they underwent severe reduction in population size recently^3^, the effective population size estimated was considerably higher (Fig. 3a), this is possibly due to the limitation of PopSizeABC method, which can overestimate effective population size when there is a large decline in population within the last few hundred years^45^, so only the trend in historical effective population size can be confidently estimated by this method. JH, TB, and PZ horses showed a common trend in population decline, starting approximately 4,500–10,000 years before present time; this was a likely consequence of horse domestication^4^.

To understand the historical relationship among the populations, we generated a maximum likelihood (ML) tree and calculated the residual covariance matrix of MH, TB, JH, and PZ using Treemix^46^ (Fig. 3b and Supplemental Fig. 2a–c). We sequentially added the migration events to the ML tree and inferred that the tree with one migration edge had the smallest residual error, therefore the best fit for our data. This tree indicated a gene flow from PZ to MH (Supplemental Fig. 2d–f). However there was no significant migration edge between the JH and MH. Archeological evidence suggests that horses were present in the Jeju island prior to the introduction of the Mongolian horses in the 12^th^ century^10^, and there are reports of size similarity between the JH and horses in southern japan^47^, and phylogenetic analysis based on mitochondrial genome sequence had placed the JH with European and Middle eastern breeds^3^ rather than the Mongolian breeds, these results along with our findings, leads us to speculate that the JH has an ancestry independent of the MH, however since 2 of the 12 JH samples used in this study showed some admixture with MH, a future study with a larger number of JH samples must be carried out, to verify if there is a population substructure within the JH, one with no admixture with Mongolian horse and another with admixture.

### Signature of selection in the genome of the Jeju horse

**Figure 4:**
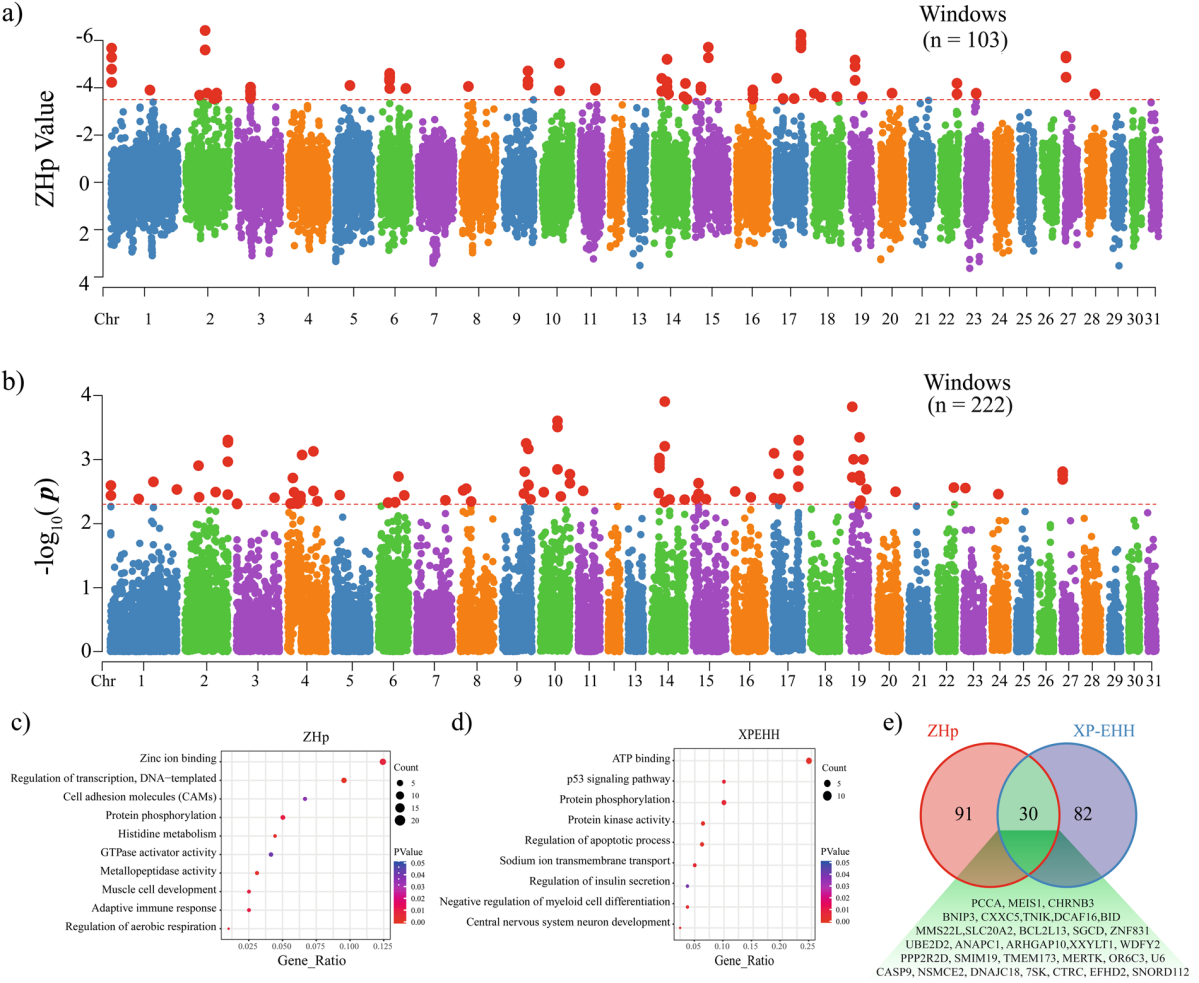
Selection signature analysis. (**a,b**) Manhattan plot showing the distribution of *P* values inferred from ZHp **(a)** and XP-EHH **(b)** analyses. The XP-EHH analysis was performed between JH and TB horses, while ZHp was performed only with JH. The total number of windows found to be significant is provided at the top of the panel. **c - d)** Enriched gene ontology and KEGG pathway for genes in the candidate selection signature regions in ZHp (c) and XP-EHH (d). (**e**) Venn diagram showing the total number of genes found within the significant candidate selection signature regions.

The characteristic features of the JH are its small body size, stocky head and shorter limbs, they also have poor athletic ability, in comparison, the TB horse are tall, slender and have been intensively artificially selected for their athletic ability, and candidate genes underlying body confirmation athletic performance traits are very well characterized^17,18,20,48–50^. Therefore, we performed a cross-population extended haplotype homozygosity (XP-EHH)^51^ between the JH and TB genome to identify selection signatures for body confirmation and performance traits in JH, and also looked for signatures for environmental adaptation within the JH genome by examining heterozygosity and extreme haplotype homozygosity using Z transformations of pooled heterozygosity (ZHp)^52^. First, we scanned the whole genome to detect regions with high degrees of fixation, which are indicative of selection signatures^53^. We applied 50% overlapping sliding windows, which were 150 Kb in size, along all the 31 autosomes. ZHp scores were calculated for each of the 29,840 windows analyzed. The ZHp scores ranged from −6.50 to 3.63 in the JH (Fig. 4a and Supplemental file S8). Because an extremely low ZHp score indicates putative selective sweep due to excess homozygosity, we focused on ZHp scores in the extreme lower end of the distribution. We observed 103 windows with ZHp scores of less than −3.5 in the JH. GO analysis of the candidate selection signature genes showed significant enrichment (P < 0.05) for adaptive immune response, muscle cell development, regulation of aerobic respiration, zinc ion binding, and cell adhesion molecules (Fig. 4c).

Next, we divided the whole genome into non-overlapping windows of 50 Kb. A total of 43,061 windows were used to calculate the XP-EHH in each window. We observed 222 windows with significant XP-EHH (P ≤ 0.005) in the JH (Fig. 4b). These windows were considered candidate regions under selection and used for further analyses. As shown by the distribution of raw ZHp and XP-EHH values in Fig. 4a,b, we observed 121 and 112 genes in the candidate regions for ZHp and XP-EHH, respectively. GO analysis showed significant enrichment (P < 0.05) for ATP binding, p53 signalling pathway, and protein kinase activity (Fig. 4d). Among the candidate regions evaluated using these two approaches only 30 genes overlapped, they were enriched for apoptotic processes, mitochondrial outer membrane permeabilization, signal transduction in response to DNA damage, and mitotic nuclear division (Table 6 and Fig. 4e). The overlapping of low number of genes may be due to differences in the statistical methods used for data analysis. Haplotype-based selection signature analysis, such as XP-EHH, has a greater power, to detect ongoing selection, while analyses based on allele frequency patterns, such as ZHp, can detect recent fixation of alleles^54^. The candidate selection signature genes, indicates selection for environmental adaptation, athletic performance, muscle cell development and body confirmation traits.

**Table 6:** Gene ontology analysis of genes that overlapped between the two selection signature analysis methods.

GO_ID	Term	Count	Genes	*P* value
GO:0034349	Glial cell apoptotic process	2	BID, CASP9	0.007127
GO:0097345	Mitochondrial outer membrane permeabilization	2	BID, BNIP3	0.011379
GO:0042770	Signal transduction in response to DNA damage	2	BID, CASP9	0.015615
GO:0090200	Positive regulation of release of cytochrome c from mitochondria	2	BID, BNIP3	0.039287
GO:0006915	Apoptotic process	5	TMEM173, CASP9, BNIP3, BCL2L13,BID	0.045793
GO:0007067	Mitotic nuclear division	3	ANAPC1, NSMCE2, PPP2R2D	0.048449

### Genes underlying physical conformation traits in the Jeju horse

Among the regions under selection, we identified several genes associated with equine body confirmation traits such as body size; wither height, and dwarfism (Table 5). These included *LCORL*, *HMGA2*, *NCAPG*, *PROP1*, and *ZFAT*. The ligand-dependent nuclear receptor co-repressor like protein (*LCORL*) is associated with body size in several horse breeds^18,55–57^. Moreover, a recent study has shown that a T homozygous genotype on rs68603064 at ECA 3 near *LCORL* is significantly associated with lower wither height in Brazilian pony, with average wither height less than 158 cm^58^. In this study, we observed the same SNP to be fixed in the JH, which shows an average wither height of 122 cm. This observation was consistent with the previous results of Schroder *et al*. (2010), who found that a QTL on ECA 3 in the region of *LCORL* influences body conformation traits such as head, neck, and frame development^57^. Notably, the JH also shows a heavy head, thick neck, and short and thick limbs^59^.

A large-scale genome-wide analysis, examining 65 horse breeds, identified 4 loci on ECA 3, 6, 9, and 11 near *LCORL*/*NCAPG*, *HMGA2*, *ZFAT*, and *LASP1* to account for the 83% variation in body size in horses^56^. These four genes (*LCORL*, *HMGA2*, *NCAPG*, and *ZFAT*) were present in the candidate selection signature regions in JH. Zinc finger and AT hook domain containing gene (*ZFAT*) on ECA 9 may play an important role in haematopoiesis^60^ and may be associated with height in horses^55,56^. The horse *ZFAT* gene is orthologous to human *ZFAT*, which influences height^61–63^. The four SNPs (rs68748127, rs68750453, rs68748129, rs68748130) in the *ZFAT* gene were fixed in the JH (Table 5). The high mobility group AT-hook 2 gene (*HMGA2*) is an architectural transcription factor that regulates and directs cellular growth, proliferation, and differentiation^64^. Moreover, *HMGA2* is amongst the first genes associated with human height^61,65^. *HMGA2* plays a role in the control of body size in dogs^66,67^. Prophet of the Pit-1 gene (*PROP1*) is a homeodomain transcription factor associated with pituitary development^68^. Mutations in this gene are implicated in deficiency of pituitary hormone in humans^69^. *PROP1* causes dwarfism in Ames dwarf mice^70^ and may be associated with dwarfism in Friesian horses^71^. The presence of these genes in candidate selection signature regions indicates that these genes may robustly drive selection for body confirmation traits and small body size in JH. These genes are also good candidates for further targeted studies to find the causative SNPs associated with physical traits and small body size in horses.

### Genes under selection for athletic performance and muscle fibre composition

**Figure 5:**
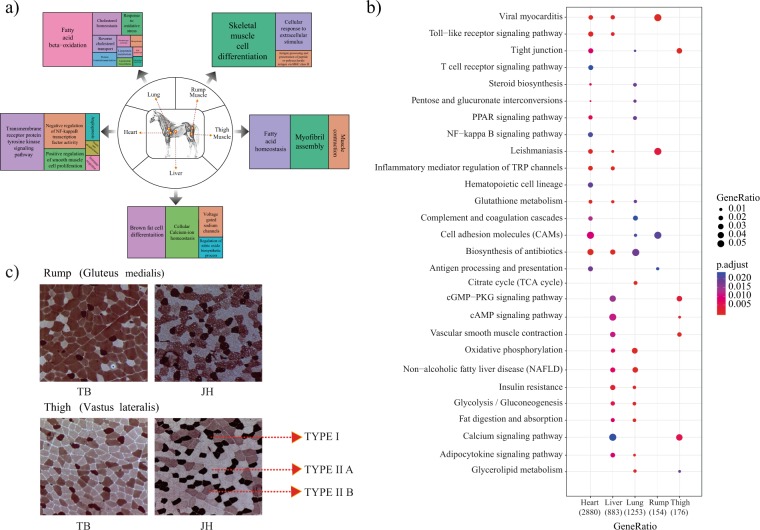
Results of RNAseq analysis of JH and TB. (**a,b**) Summary of gene ontology (**a**) and KEGG pathways (**b**) enriched in the five tissues acquired from JH. (**c**) Muscle fibre composition analysis form rump and thigh muscle biopsy in JH and TB population.

The JH is an indigenous native horse breed predominantly used for draught purpose in the nomadic habitual environment, while TB has been intensively selected for superior athletic performance. Because these two breeds are intended for different purposes, comparison of the genomes revealed numerous genes associated with athletic performance and muscle composition. Muscle fibres are classified into Type I (slow twitch), and Type IIA, IIB, and IIC (fast twitch)^72^. Horses with higher content of slow-twitch oxidative muscle fibres develop strength and power, and are well-suited for draught work and meat production. The muscles of horses selected for racing, high speed running, and other athletic purposes consist of fast-twitch glycolytic fibres^73^. Comparative histochemical analysis of gluteus medialis (rump muscle) and vastus lateralis (thigh muscle) in the JH and TB showed that the content of Type I fibres in the JH exceeded that in TB by ~15%–20% (Fig. 5C, Supplemental Figure S3).

In the candidate selection signature regions, we identified five genes that are associated with athletic performance, namely, *ACTN2*^50^, *COX4I1*^50^, *COX4I2*^49^, *CKM*^49^, and *PDK4*^50^. Creatine kinase muscle (*CKM)*, which is a muscle type isozyme of creatine kinase, and *COX4I2*, which is a subunit of cytochrome c oxidase (*COX*), increase the efficiency of cellular respiration and are associated with athletic performance in TB^49^. Similarly, pyruvate dehydrogenase kinase isozyme 4 (*PKD4*) plays a key role in oxidation of fatty acids for ATP generation^49^. Three SNPs on *PKD4* (g.39020227, g.39017959, and g.39024151) are significantly associated with winning performance in elite TB horses^50^. In particular, the A alleles of both g. 39017959 C > A and g. 39024151 G > A were found to be the favourable allele at both loci and were strongly associated with athletic performance TB, the *C* and *G* alleles were found to be fixed at the respective loci in the JH. Similarly, g.16079732 A > G (*CKM*) and g.23314524 C > T (*COX4I2*) were also associated with athletic performance in TB, with the *A* and *T* being the favourable alleles^49^. However, the *C* allele of g. 23314524 > T was fixed in JH, and the *G* allele of g. 16079732 A > G was the predominant allele (0.90) in JH (Table 5). The two genes *ACTN3* and *MSTN*, which function in muscle development and muscle fibre composition, were also identified in the selection signature regions. *ACTN3* is a sarcomeric protein involved in structure, muscle metabolism, and calcineurin signalling^74,75^. Mutations in *ACTN3* change muscle fibre phenotype from fast-twitch fibres to slow-twitch fibres^75^. Moreover, rs1144978872 in the 5′UTR of *ACTN3* leads to g.30226577 G > A and may affect athletic performance^74,75^. Interestingly, the *A* allele of g.30226577 G > A was identified as being detrimental to sprint performance, while endowing the carrier with strength and power suitable for draught work. The *A* allele (0.89) and Type I fibres predominated in the JH (Table 5).Myostatin, encoded by the *MSTN* gene, limits skeletal muscle mass by controlling and regulating the growth and number of muscle fibres^76–78^. Variations in *MSTN* are implicated in muscle fibre composition in horses^73,79–81^. The presence of a short interspersed nuclear element (SINE; 227 bp) in the promoter region and *C* allele in intron 1 (Chr 18: 66608679; rs397152648) are significantly associated with a greater proportion of Type 2B fibres and reduction in Type I fibres in TB and Quarterback horse populations^80,81^. The promoter SINE was absent, while the variant *T* allele at g.66608679was highly predominant (0.9) in the JH. Moreover, the g. 66608679 C > T on *MSTN* is also associated with sprinting ability and distance racing, and is known to be a gene associated with speed^48^. The *C* allele may be better suited for fast and short-distance races, while the *T* allele is suited for long-distance slower-speed racing^82^. Hence, the predominant *T* allele (0.95) in the JH may explain the ability of this breed to run long distances and its strong endurance in the nomadic habitual environment.

The majority of identified candidate selection signature genes for body confirmation, athletic performance and muscle compositions were identified in comparison with the TB horse genome (XP-EHH method). Since the TB horse are artificially selected for these traits, they could be representative of this selection, therefore the functional variants and genes identified as selection signatures in JH must be validated by further genetic studies such as genome-wide association studies.

### Transcriptomic landscape of the JH compared with that of TB

To understand the genetic basis underlying the morphological and physiological differences in body confirmation and running abilities of JH relative to TB horses, we compared mRNA expression levels between JH and TB, in five tissues including the rump muscle, thigh muscle, liver, heart, and lung. These five tissues were chosen for their critical role in energy metabolism, aerobic capacity and athletic ability of horse. The RNAseq analysis showed that relative to TB, 5,462 genes were found to be differentially expressed (DE) in JH, including 3,498 genes in heart, 1,101 genes in the liver, 1,417 genes in the lung, 139 genes in rump muscle, and 193 genes in thigh muscle (Supplemental file S9). GO analysis (Fig. 5a) and KEGG pathway analysis (Fig. 5b), conducted using DEGs, indicated significant enrichment in the following terms: transmembrane receptor kinase signalling pathway; positive regulation of smooth muscle cell proliferation; immune response; angiogenesis and cell adhesion molecules (CAM) pathway; Toll-like receptor signalling pathway and tight junction pathways in heart; skeletal muscle cell differentiation; cellular response to extracellular stimulus and CAM pathway in the rump muscle; fatty acid homeostasis; muscle contraction and calcium signalling pathway in the thigh muscle; brown fat cell differentiation; cellular calcium ion homeostasis; oxidative phosphorylation pathway and biosynthesis of antibiotics pathway in the liver; fatty acid beta oxidation; cholesterol homeostasis; response to oxidative stress; cAMP signalling pathway; fat digestion and absorption pathway; and calcium signalling pathway in the lung.

Among the DE genes, 71 genes were located in the candidate selection signature regions (Supplemental file S10) identified in the JH. The majority of these genes were in the heart (39 genes), followed by liver (16 genes), lung (16 genes), rump (3 genes), and 1 gene in the thigh muscle. Five of these genes (*LCORL*, *snoR442*, *GRIA2*, *PRRT1*, and *U6atac*) were up-regulated in all these tissues; 4 genes (*U6*, *U4*, *SNORD112*, and *7SK*) were up-regulated in the liver and down-regulated in the heart; and the rest of the genes were down-regulated in all these tissues. Interestingly *LCORL*, a gene associated with the body size of horse was differentially expressed. A previous study has shown that in hanoverian wamblood horses the expression level of *LCORL* was substantially higher in the *TT* horse (rs68603064), which has a smaller body size, than in *CT* and *CC* horses^18^. We found the *T* allele to be fixed in the JH at the rs68603064 locus. Our result also showed the expression of *LCORL* was significantly higher in the JH (FDR < 0.05) than in the TB horse. Several other important genes responsible for muscle fibre composition (*MSTN*) and athletic performance (*ACTN2, COX4I1*, *COX4I2*, and *CKM*) were downregulated in JH tissues. Long-term athletic endurance requires high oxygen carrying capacity (aerobic capacity; Vo_2_max), high density of mitochondria in skeletal muscle, and a large lung volume^83,84^. The abundance of mitochondrial *COX4* (*COX4 I1 and COX4I2*) is directly related to mitochondrial density^85^. The expression of *COX4I1* and *COX4I2* can also affect velocity at maximum heart rate (VHRmax). VHRmax is a predictor for overall athletic ability and peak post-exercise plasma lactate concentration; consequently VHRmax is generally used as an essential predictor for overall anaerobic capacity of TB^83,84^. In TB that are bred for superior athletic ability, *COX4I1* levels are significantly increased after exercise, leading to a positive correlation with VHRmax^86^. Therefore, the significant downregulation of *COX4I1* and *COX4I2* expression observed in the JH may indicate a lower oxygen carrying capacity in the JH, resulting in its reduced anaerobic capacity and ability for athletic racing. However, this requires further study, including measuring and comparing lung capacity, VHRmax, and Vo_2_max between JH and TB horses.

In summary, we identified differential expression of numerous genes within the candidate selective sweep regions, indicating that the extensive adaptation of the JH conferred it with physiological and morphological variation relative to that of TB. These results helps in understanding the unique adaptations found in the JH.

There are limitations in this study due to the use of only TB (a highly artificially selected breed for athletic performance) as a reference for compassion with JH for identifying selection signature and gene expression difference. Further studies involving other extreme sized horse breeds such as small sized: shetland ponies, highland ponies, and large sized: shire horses, clydesdales and belgian draft horses could help in identifying regions under selection and genes that are differentially expressed in JH for stature, hardiness, longevity, muscle composition and strength.

## Conclusions

In this study, we generated whole-genome sequence data on 41 horses that included three MH breeds (MG, MJ, and MD), as well as PH, TB, and JH. We identified 5.1 million (~24%) novel SNPs among the ~21 million observed SNPs. Additionally, we identified breed-specific nsSNPs and LOF mutants in the JH and TB, respectively. Analyses performed using data on population genetics, admixture, and demography indicated that the JH did not genetically originate from MH breeds, even though the JH is closer to MH genetically than to other horse breeds examined in this study. We also found that nucleotide diversity in the PZ, which was close to extinction in the last century, is much higher than that in the JH, indicating lower genetic diversity within JH and the need to design conservation strategies to increase and maintain genetic diversity within the JH population. Our selection-signature analyses, based on allele frequency homozygosity and haplotype fixation, revealed numerous candidate genes involved in the environmental adaptation, muscle composition, and unique body confirmation traits of the JH. Finally, we performed RNAseq analysis using five types of tissues (heart, lung, liver, rump muscle, and thigh muscle) acquired from the JH and TB, and found numerous important DE genes within the selective sweep regions. The data generated in this study will serve as a valuable resource for researchers studying the evolution and domestication of *Equus caballus*; these data can also be used for the study of other small-sized or non-bred horse breeds. The large number of novel SNPs identified in this study will aid in designing a genome-wide high-density SNP chip. This will help to design conservation strategies for various native horse breeds across the globe.

## Methods

### Sampling and whole-genome re-sequencing

All the experimental procedures were verified and approved by the Institutional Animal Care and Use committee of the National Institute of Animal Science (NIAS2015–775), and all methods were performed in accordance with the relevant guidelines and regulations.

Whole-genome re-sequencing data were generated for Mongolian Galshar (N = 6, MG), Mongolian Jarglant (N = 5, MJ), Mongolian Domestic Horses (N = 4, MD), Przewalski’s Horses (N = 4, PZ), Jeju horse (N = 12, JH), and Thoroughbred horse (N = 10, TB) (Table 1). Samples of JH and TB tissues were obtained from the National Institute of Animal Science (NIAS, Jeju Island, Korea). Samples of MH tissues were obtained from Mongolian University of Life Sciences (Ulaanbaatar, Mongolia). PZ horse samples were obtained from Seoul Zoo (Seoul, Republic of Korea). Indexed shotgun paired-end libraries with average insert size of 500 bp were generated using TruSeq Nano DNA Library Prep Kit (Illumina, San Diego, CA, USA) following the standard Illumina sample-preparation protocol. Briefly, 200 ng of gDNAs were fragmented with Covaris M220 (Woburn, MA, USA) to obtain median fragment size of ~500 bp. These fragmented DNAs were end-repaired, followed by A-tailing and ligation to the indexed adapter (~125-bp adapter). Gel-based selection was performed to select sizes of 550 to 650 bp. Eight cycles of PCR amplification was performed on GeneAmp PCR system 2700 thermal cycler (Applied Biosystems®, Foster City, CA). Size-selected libraries were then analysed with Agilent Bioanalyzer 2100 (Agilent, Santa Clara, CA, USA) to determine size distribution and adapter contamination status. The resulting libraries without adaptor contamination were sequenced on Illumina HiSeq. 4000 sequencing platforms for 2 × 125 bp paired-end sequencing.

### Sequence mapping, SNP calling, and annotation

After quality-control assessment and trimming, the sequences were aligned to Equine reference genome assembly (*EquCab* 2.0^34^) using Bowtie2 v2.2.4^87^ at default parameters. Mapped reads were converted, sorted, and indexed using SAM-tools v1.3.1^88^. Removal of duplicate reads and generation of quality matrices for mapping were performed using Picard tools v2.1.0 (http://broadinstitute.github.io/picard). Local recalibration and realignment were conducted using Genome Analysis Toolkit (GATK; v3.3). A multi-sample SNP-calling procedure was performed to discover SNPs using UnifiedGenotyper in the GATK package^89^. Finally, a filtering step, based on GATK best practice guidelines was used as follows: QD < 5.0, MQ < 40.0, FS > 200.0, and QUAL < 30.0^89^. SNPs were also filtered for call rate < 0.9 and MAF (minor allele frequency) ≤ 0.01.The filtered SNPs were then annotated to 12 functional categories (Table 2) using SnpEff version 4.1^41^. SnpSift version 4.1 was used for filtering loss-of-function (LOF) and non-synonymous (NS) mutations. We also determined which SNPs were fixed in the JH and TB, and identified breed-specific fixed NS mutants. Non-reference genotypes that were homozygous for the entire population were deemed fixed SNPs^90^.

### Remapping of the identified SNPs to EquCab3.0 Coordinates

Following the release of the new reference genome *EquCab3.0*^91^ we remapped the coordinates of the SNPs identified using the *EquCab* 2.0 (Assembly SeqID GCF_000002305.2) reference genome to the corresponding *EquCab* 3.0 (Assembly SeqID GCF_002863925.1) coordinates using the NCBI Remap tool (May 2018 release), which facilitates the remapping of genomic features from one assembly to another. All options were set to default. Only *EquCab 3.0* annotated SNPs are reported and discussed herein. And all the genomic coordinates given are based on *EquCab* 3.0.

### Population genetics analysis

VCFtools v4.0^92^ was used to estimate mean autosomal nucleotide diversity using windows of 10 kb. PCA analysis was performed using genome-wide complex trait analysis (GCTA)^93^. Genotype data for all the samples were used to estimate the eigenvectors. To refine the quantification of different ancestry proportions, we performed a model-based unsupervised hierarchical clustering of the individuals using Admixture 1.22 software^44^. Admixture provides a likelihood estimate in which ancestral populations in an unsupervised analysis are clustered based on allele-frequency similarities. We analysed breed proportions using K = 2 to 6 assumed ancestral populations. Recent demographic history was inferred by assessing changes in the trend of effective population size (Ne) using PopSizeABC^94^ with parameters set as described previously^35^. An IBS-based distance matrix was calculated using SNP genotypes in PLINK v1.9^95^, and a neighbour-joining tree was constructed with FigTree v1.4.4. We then inferred population-level phylogeny based on maximum likelihood (ML) statistics implemented in TreeMix^46^. A linkage disequilibrium (-k) size of 1000 and ‘-global’ options were used to generate the ML tree, and migration events (-m) were sequentially added to the tree.

### Selective sweep analysis

We used Z transformation of pooled heterozygosity (ZHp) and XP-EHH to detect putative selective sweeps (positive selection) in the JH population. All the high-quality SNPs, derived from the JH, were used to identify pooled heterozygosity (ZHp). The numbers of major and minor alleles were counted; then, SNP positions with a minor allele frequency less than 0.05 were removed. Subsequently, we applied 50% overlapping sliding windows that were 150 kb is size; ZHp was eventually calculated for each window after removing windows with fewer than 10 SNPs following the method by Rubin *et al*.^52,96^. A threshold of 3 was used for identifying significant signals. We then used xpehh software (http://hgdp.uchicago.edu/Software/) to perform a cross-population extended haplotype homozygosity (XP-EHH) analysis to detect selection signatures in the JH population relative to those in the thoroughbred horse population^51^. This analysis detects haplotypes that show increased frequency to the point of complete fixation in one of the populations. We initially split the genome into non-overlapping segments of 50 kb and used the maximum (positive) XP-EHH score of all SNPs within a window as a summary statistic for that window. To assess variations in SNP density, we binned genomic windows according to their numbers of SNPs in increments of 500 SNPs (combining all windows ≥ 1000 SNPs into one bin). Within each bin, for each window i, the fraction of windows with a value of the statistic greater than in i was defined as the empirical *P* value. Regions with *P* value less than 0.005 (0.5%) were considered statistically significant signals. Regions that were found to be significant using either one of the methods were considered as candidate regions under selection (CRS).

### RNAseq analysis

Total RNA from five types of tissues (heart, lung, liver, rump muscle, and thigh muscle) was isolated from JH (n = 3) and TB (n = 4) using Trizol reagent (Invitrogen, Carlsbad, CA, USA) following the manufactures protocol. The quality of isolated RNA was assessed using Bioanalyzer 2100 (Agilent, Santa Clara, CA, USA). Only RNA with an RNA integrity value (RIN) greater than 8 was used for library preparation. The sequencing libraries were prepared using a TruSeq RNA kit (Illumina; San Diego, CA), and Paired End sequencing was carried out on an Illumina HiSeq-2000 (Illumina; San Diego, CA), using individual lanes. The generated reads were trimmed to remove adapter sequences using Trimmomatic 0.36^97^; the reads were assessed for quality using FastQC version 0.11.6^98^. The reads were then aligned to the Equine reference genome assembly (EquCab2.1) using Hisat2 version 2.05^99^. The aligned reads were counted using featureCounts in the Subread package version 1.6.0^100^. The count data were analysed for differential gene expression (DEG) using EdgeR package in R^101^. Genes with a false discovery rate corrected *Q* value < 0.05 were considered to be significantly differentially expressed and were used for downstream analysis.

### Functional enrichment analysis

Functional enrichment analysis was performed on genes in the CRS, genes with LOF and NS mutations, and differentially expressed genes (DE). The enriched GO terms were obtained using the web program DAVID (Databank for Annotation, Visualization and Integrated Discovery)^102^; REVIGO^103^ and Clusterprofiler R package^104^ were used for summarizing the GO terms.

### Histochemical analysis

Histochemical analysis was performed to examine muscle fibre characteristics in the gluteus medialis (rump) and vastus lateralis (thigh) muscles of JH and TB horses. The analysis was performed as previously described by Lee *et al*.,^105^. Briefly, tissue samples were collected from two horses per breed. These samples were cut into 0.5 cm * 0.5 cm * 1.0 cm blocks, frozen in isopentane cooled by liquid nitrogen, and stored at −80 ^◦^C until further use. The muscle tissues were consequently sectioned at 10μm thickness using a cryostat microtome (CM 1850, Leica, Heidelberger, Germany) at 20^◦^C, and the activity of myosin adenosine triphosphatase (ATP) was detected after acid pre-incubation (pH 4.7) as described previously^106^. The predominance of Type I or Type II muscle fibres in the two breeds was then evaluated using an optical microscope, equipped with a charge-coupled device (CCD) colour camera (IK-642K, Toshiba, Tokyo, Japan), and image analysis was carried out using Image-Pro plus software (Media,Cybernetics, Silver Springs, USA).

## Supplementary information

Supplementary Figures

Supplemental file S1

Supplemental file S2

Supplemental file S3

Supplemental file S4

Supplemental file S5

Supplemental file S6

Supplemental file S7

Supplemental file S8

Supplemental file S9

Supplemental file S10

## Data Availability

All the data generated in this study are freely available for download at the National Agricultural Biotechnology Information Center (NABIC) website (www.nabic.rda.go.kr). The accession numbers for whole genome data are JH (NN-5490-000001 ~ NN-5490-000012), TB(NN-5491-000001 ~ NN-5491-000010), MH and PZ (NN-5492-000001 ~ NN-5492-000017), and for RNAseq data are JH (NN-5494-000001 ~ NN-5494-000015), TB (NN-5495-000001 ~ NN-5495-000019).

## References

[1] Jo Y-S, Kim T-W, Choi B-J, Oh H-S (2012). Current status of terrestrial mammals on Jeju Island. Journal of Species Research.

[2] Kim B-W (2011). Characterization of the European type of maternal lineage evident in extant Jeju native pigs. Genes & Genomics.

[3] Yoon SH (2017). Complete mitochondrial genome sequences of Korean native horse from Jeju Island: uncovering the spatio-temporal dynamics. Molecular biology reports.

[4] Levine M (1999). Investigating the origins of horse domestication. Equine Veterinary Journal.

[5] Vilà C (2001). Widespread origins of domestic horse lineages. Science.

[6] Kim, N. Y. *et al*. Genome-wide analyses of the Jeju, Thoroughbred, and Jeju crossbred horse populations using the high density SNP array. *Genes & genomics*, 1–10 (2018). 10.1007/s13258-018-0722-0 30099720

[7] Do K-T, Lee J-H, Lee H-K, Kim J, Park K-D (2014). Estimation of effective population size using single-nucleotide polymorphism (SNP) data in Jeju horse. Journal of animal science and technology.

[8] Lee J-H, Song K-D, Kim J-M, Leem H-K, Park K-D (2016). Identification of genes with nonsynonymous SNP in Jeju horse by whole-genome resequencing reveals a functional role for immune response. Journal of animal science.

[9] Nam D (1969). Horse production in Cheju during Lee dynasty. Studies on Korean History.

[10] Shin T (1992). An anatomy study of animal bones excavated in the Kwakji archaeological site in Cheju Island. Go-Moon-Wha.

[11] Kim KI (1999). Phylogenetic relationships of Cheju horses to other horse breeds as determined by mtDNA D‐loop sequence polymorphism. Animal Genetics.

[12] Oh, M. *et al*. Phylogenetic relationship of Cheju native horses by mitochondrial DNA analysis. *Molecules and Cells**(Korea Republic)* (1994).

[13] Xu S (2007). High altitude adaptation and phylogenetic analysis of Tibetan horse based on the mitochondrial genome. Journal of Genetics and Genomics.

[14] Jung Y-H, Han S-H, Shin T, Oh M-Y (2002). Genetic characterization of horse bone excavated from the Kwakji archaeological site, Jeju, Korea. Molecules and cells.

[15] Yang Y, Kim K, Cothran E, Flannery A (2002). Genetic diversity of Cheju horses (Equus caballus) determined by using mitochondrial DNA D-loop polymorphism. Biochemical genetics.

[16] Kim NY (2018). Estimation of genetic parameters for temperament in Jeju crossbred horses. Asian-Australasian journal of animal sciences.

[17] Brown-Douglas, C. G. & Pagan, J. D. Body weight, wither height and growth rates in Thoroughbreds raised in America, England, Australia, New Zealand and India. *Advances in Equine**Nutrition IV*, 213 (2009).

[18] Metzger J, Schrimpf R, Philipp U, Distl O (2013). Expression levels of LCORL are associated with body size in horses. PLoS One.

[19] Lee J-R (2014). Genome-wide analysis of DNA methylation patterns in horse. BMC genomics.

[20] Lee W (2018). Analysis of cross-population differentiation between Thoroughbred and Jeju horses. Asian-Australasian journal of animal sciences.

[21] Choi S-K, Cho C-Y, Yeon S-H, Cho B-W, Cho G-J (2008). Genetic characterization and polymorphisms for parentage testing of the Jeju horse using 20 microsatellite loci. Journal of Veterinary Medical Science.

[22] Gim J-A (2015). HEpD: A database describing epigenetic differences between Thoroughbred and Jeju horses. Gene.

[23] Schubert M (2014). Prehistoric genomes reveal the genetic foundation and cost of horse domestication. Proceedings of the National Academy of Sciences.

[24] Do K-T (2014). Genomic characterization of the Przewalski׳ s horse inhabiting Mongolian steppe by whole genome re-sequencing. Livestock Science.

[25] Goto H (2011). A massively parallel sequencing approach uncovers ancient origins and high genetic variability of endangered Przewalski’s horses. Genome biology and evolution.

[26] Huang J (2014). Analysis of horse genomes provides insight into the diversification and adaptive evolution of karyotype. Scientific reports.

[27] Volf, J., Kus, E. & Prokopova, L. General studbook of the Przewalski horse. *Zoological Garden Prague, Prague* (1991).

[28] Minjigrorj N, Austbo D (2009). Production of mare’s milk in Mongolia. *Billige M., Liu W., Rina W., Wang L., Sun T., Wang J., Li H., & Zhang H. Evaluation of potential probiotics properties of the screened Lactobacilli isolated from home-made koumiss in Mongolia*. Annals of Microbiology.

[29] Zhang C (2018). Detecting the Population Structure and Scanning for Signatures of Selection in Horses (Equus caballus) From Whole-Genome Sequencing Data. Evolutionary Bioinformatics.

[30] Gouveia JJdS, Silva MVGBd, Paiva SR, Oliveira SMPd (2014). Identification of selection signatures in livestock species. Genetics and molecular biology.

[31] Avise JC, Ayala FJ (2009). In the light of evolution III: Two centuries of Darwin. Proceedings of the National Academy of Sciences.

[32] Driscoll CA, Macdonald DW, O’Brien SJ (2009). From wild animals to domestic pets, an evolutionary view of domestication. Proceedings of the National Academy of Sciences.

[33] Gregory TR (2009). Artificial selection and domestication: modern lessons from Darwin’s enduring analogy. Evolution: Education and Outreach.

[34] Wade C (2009). Genome sequence, comparative analysis, and population genetics of the domestic horse. Science.

[35] Kim J (2017). The genome landscape of indigenous African cattle. Genome biology.

[36] Moon S (2015). A genome-wide scan for selective sweeps in racing horses. Asian-Australasian journal of animal sciences.

[37] Choi J-W (2014). Whole-genome analyses of Korean native and Holstein cattle breeds by massively parallel sequencing. PloS one.

[38] Consortium GP (2012). An integrated map of genetic variation from 1,092 human genomes. Nature.

[39] Choi J-W (2015). Whole-genome resequencing analyses of five pig breeds, including Korean wild and native, and three European origin breeds. DNA Research.

[40] Wang J, Raskin L, Samuels DC, Shyr Y, Guo Y (2014). Genome measures used for quality control are dependent on gene function and ancestry. Bioinformatics.

[41] Cingolani P (2012). A program for annotating and predicting the effects of single nucleotide polymorphisms, SnpEff: SNPs in the genome of Drosophila melanogaster strain w1118; iso-2; iso-3. Fly.

[42] Kaiser VB (2015). Homozygous loss-of-function variants in European cosmopolitan and isolate populations. Human molecular genetics.

[43] Oh HJ, Choi D, Goh CJ, Hahn Y (2015). Loss of gene function and evolution of human phenotypes. BMB reports.

[44] Alexander DH, Novembre J, Lange K (2009). Fast model-based estimation of ancestry in unrelated individuals. Genome research.

[45] Boitard S, Rodriguez W, Jay F, Mona S, Austerlitz F (2016). Inferring population size history from large samples of genome-wide molecular data-an approximate Bayesian computation approach. PLoS genetics.

[46] Pickrell JK, Pritchard JK (2012). Inference of population splits and mixtures from genome-wide allele frequency data. PLoS genetics.

[47] Nozawa K, Kondo K (1970). Gene constitution of Cheju native horse and its phylogenetic relationships with Japanese native horses. SABRAO Newsletter.

[48] Hill, E. W. *et al*. Correction: A sequence polymorphism in MSTN predicts sprinting ability and racing stamina in thoroughbred horses. *PloS one*, **5** (2010). 10.1371/journal.pone.0008645 PMC2808334 20098749

[49] Gu J (2010). Association of sequence variants in CKM (creatine kinase, muscle) and COX4I2 (cytochrome c oxidase, subunit 4, isoform 2) genes with racing performance in Thoroughbred horses. Equine Veterinary Journal.

[50] Hill E, Gu J, McGivney B, MacHugh D (2010). Targets of selection in the Thoroughbred genome contain exercise‐relevant gene SNPs associated with elite racecourse performance. Animal genetics.

[51] Sabeti PC (2007). Genome-wide detection and characterization of positive selection in human populations. Nature.

[52] Rubin C-J (2012). Strong signatures of selection in the domestic pig genome. Proceedings of the National Academy of Sciences.

[53] Qanbari S (2012). A high resolution genome-wide scan for significant selective sweeps: an application to pooled sequence data in laying chickens. PloS one.

[54] Qanbari S, Simianer H (2014). Mapping signatures of positive selection in the genome of livestock. Livestock Science.

[55] Signer-Hasler H (2012). A genome-wide association study reveals loci influencing height and other conformation traits in horses. PloS one.

[56] Makvandi-Nejad S (2012). Four loci explain 83% of size variation in the horse. PLoS One.

[57] Schröder, W. Athletic performance and conformation in Hanoverian warmblood horses-population genetic and genome-wide association analyses. cumulative thesis. *Hannover: University of Veterinary Medicine* (2010).

[58] Junior AB (2018). Polymorphisms in the LASP1 gene allow selection for smaller stature in ponies. Livestock Science.

[59] Kang M-s (2004). Grazing behaviour of Jeju Native Horses. Journal of integrated field science.

[60] Tsunoda T (2010). Immune-related zinc finger gene ZFAT is an essential transcriptional regulator for hematopoietic differentiation in blood islands. Proceedings of the National Academy of Sciences.

[61] Allen HL (2010). Hundreds of variants clustered in genomic loci and biological pathways affect human height. Nature.

[62] Takeuchi F (2009). Evaluation of genetic loci influencing adult height in the Japanese population. Journal of human genetics.

[63] N’Diaye A (2011). Identification, replication, and fine-mapping of Loci associated with adult height in individuals of african ancestry. PLoS genetics.

[64] Cleynen I, Van de Ven WJ (2008). The HMGA proteins: a myriad of functions. International journal of oncology.

[65] Weedon MN (2007). A common variant of HMGA2 is associated with adult and childhood height in the general population. Nature genetics.

[66] Boyko AR (2010). A simple genetic architecture underlies morphological variation in dogs. PLoS biology.

[67] Jones P (2008). Single-nucleotide-polymorphism-based association mapping of dog stereotypes. Genetics.

[68] Davis S (2010). Molecular mechanisms of pituitary organogenesis: in search of novel regulatory genes. Molecular and cellular endocrinology.

[69] Deladoëy J (1999). “Hot spot” in the PROP1 gene responsible for combined pituitary hormone deficiency. The Journal of Clinical Endocrinology & Metabolism.

[70] Sornson MW (1996). Pituitary lineage determination by the Prophet of Pit-1 homeodomain factor defective in Ames dwarfism. Nature.

[71] Orr N (2010). Genome‐wide SNP association–based localization of a dwarfism gene in Friesian dwarf horses. Animal Genetics.

[72] Rivero J, Serrano AL, Henckel P, Aguera E (1993). Muscle fiber type composition and fiber size in successfully and unsuccessfully endurance-raced horses. Journal of Applied Physiology.

[73] Dall’Olio, S. *et al*. Analysis of horse myostatin gene and identification of single nucleotide polymorphisms in breeds of different morphological types. *BioMed Research International*, **2010** (2010). 10.1155/2010/542945 PMC2913906 20706663

[74] Thomas K, Hamilton N, North K, Houweling P (2014). Sequence analysis of the equine ACTN3 gene in Australian horse breeds. Gene.

[75] Wang J (2018). Analysis of Equine ACTN3 Gene Polymorphisms in Yili Horses. Journal of Equine Veterinary Science.

[76] McPherron AC, Lawler AM, Lee S-J (1997). Regulation of skeletal muscle mass in mice by a new TGF-p superfamily member. Nature.

[77] McPherron AC, Lee S-J (1997). Double muscling in cattle due to mutations in the myostatin gene. Proceedings of the National Academy of Sciences.

[78] Miyata H (2018). Effect of Myostatin SNP on muscle fiber properties in male Thoroughbred horses during training period. The Journal of Physiological Sciences.

[79] Rooney MF, Porter RK, Katz LM, Hill EW (2017). Skeletal muscle mitochondrial bioenergetics and associations with myostatin genotypes in the Thoroughbred horse. PloS one.

[80] Petersen JL, Valberg SJ, Mickelson JR, McCue ME (2014). Haplotype diversity in the equine myostatin gene with focus on variants associated with race distance propensity and muscle fiber type proportions. Animal genetics.

[81] Petersen JL (2013). Genome-wide analysis reveals selection for important traits in domestic horse breeds. PLoS genetics.

[82] McGivney BA (2012). MSTN genotypes in T horoughbred horses influence skeletal muscle gene expression and racetrack performance. Animal genetics.

[83] Constantinopol M (1989). Oxygen transport during exercise in large mammals. II. Oxygen uptake by the pulmonary gas exchanger. Journal of Applied Physiology.

[84] Erickson B (1994). Mechanism of reduction in alveolar-arterial PO2 difference by helium breathing in the exercising horse. Journal of Applied Physiology.

[85] Flück M (2006). Functional, structural and molecular plasticity of mammalian skeletal muscle in response to exercise stimuli. Journal of Experimental Biology.

[86] Eivers SS (2009). Alterations in oxidative gene expression in equine skeletal muscle following exercise and training. Physiological genomics.

[87] Langmead B, Salzberg SL (2012). Fast gapped-read alignment with Bowtie 2. Nature methods.

[88] Li H (2009). The Sequence Alignment/Map format and SAMtools. Bioinformatics.

[89] McKenna A (2010). The Genome Analysis Toolkit: a MapReduce framework for analyzing next-generation DNA sequencing data. Genome research.

[90] Aslam ML (2012). Whole genome SNP discovery and analysis of genetic diversity in Turkey (Meleagris gallopavo). BMC genomics.

[91] Kalbfleisch, T. S. *et al*. EquCab3, an Updated Reference Genome for the Domestic Horse. *BioRxiv*, 306928 (2018).

[92] Danecek P (2011). The variant call format and VCFtools. Bioinformatics.

[93] Yang J, Lee SH, Goddard ME, Visscher PM (2011). GCTA: a tool for genome-wide complex trait analysis. The American Journal of Human Genetics.

[94] Rodriguez, W., Jay, F., Mona, S. & Austerlitz, F. Inferring population size history from large samples of genome-wide molecular data-an approximate bayesian computation approach. *Plos Genetics*, **3**(12), 1–36 (2016) (2016). 10.1371/journal.pgen.1005877 PMC4778914 26943927

[95] Purcell S (2007). PLINK: a tool set for whole-genome association and population-based linkage analyses. The American Journal of Human Genetics.

[96] Rubin C-J (2010). Whole-genome resequencing reveals loci under selection during chicken domestication. Nature.

[97] Bolger AM, Lohse M, Usadel B (2014). Trimmomatic: a flexible trimmer for Illumina sequence data. Bioinformatics.

[98] Andrews, S. FastQC: a quality control tool for high throughput sequence data. (2010).

[99] Kim, D., Langmead, B. & Salzberg, S. (2016).

[100] Liao Y, Smyth GK, Shi W (2013). The Subread aligner: fast, accurate and scalable read mapping by seed-and-vote. Nucleic acids research.

[101] Robinson MD, McCarthy DJ, Smyth G (2010). K. edgeR: a Bioconductor package for differential expression analysis of digital gene expression data. Bioinformatics.

[102] Huang DW, Sherman BT, Lempicki RA (2008). Systematic and integrative analysis of large gene lists using DAVID bioinformatics resources. Nature protocols.

[103] Supek F, Bošnjak M, Škunca N, Šmuc T (2011). REVIGO summarizes and visualizes long lists of gene ontology terms. PloS one.

[104] Yu G, Wang L-G, Han Y, He Q-Y (2012). clusterProfiler: an R package for comparing biological themes among gene clusters. Omics: a journal of integrative biology.

[105] Lee SH, Kim J-M, Ryu YC, Ko KS (2016). Effects of Morphological Characteristics of Muscle Fibers on Porcine Growth Performance and Pork Quality. Korean journal for food science of animal resources.

[106] Brooke MH, Kaiser KK (1970). Muscle fiber types: how many and what kind?. Archives of neurology.

[107] Hansen M, Knorr C, Hall A, Broad T, Brenig B (2007). Sequence analysis of the equine SLC26A2 gene locus on chromosome 14q15→ q21. Cytogenetic and genome research.

