# Author Correction: Comprehensive genome and transcriptome analyses reveal genetic relationship, selection signature, and transcriptome landscape of small-sized Korean native Jeju horse

**DOI:** 10.1038/s41598-020-74979-w

**Published:** 2020-10-21

**Authors:** Krishnamoorthy Srikanth, Nam-Young Kim, WonCheoul Park, Jae-Min Kim, Kwon-Do Kim, Kyung-Tai Lee, Ju-Hwan Son, Han-Ha Chai, Jung-Woo Choi, Gul-Won Jang, Heebal Kim, Youn-Chul Ryu, Jin-Wu Nam, Jong-Eun Park, Jun-Mo Kim, Dajeong Lim

**Affiliations:** 1 Animal Genomics and Bioinformatics Division, National Institute of Animal Science, Rural Development Administration, Wanju, 55365 Republic of Korea; 2 Subtropical Livestock Research Institute, National Institute of Animal Science, Rural Development Administration, Jeju-do, 63242 Republic of Korea; 3 Cancer Genetics and Comparative Genomics Branch, National Human Genome Research Institute, National Institutes of Health, Bethesda, MD 20892 USA; 4 C&K genomics, Seoul, Republic of Korea; 5 Animal Breeding and Genetics Division, National Institute of Animal Science, Rural Development Administration, Wanju, 55365 Republic of Korea; 6 College of Animal Life Science, Kangwon National University, Chuncheon, 24341 Republic of Korea; 7 Division of Biotechnology, Jeju National University, Jeju, 63243 Republic of Korea; 8 Department of Life Science, Hanyang University, Seoul, 133-791 Republic of Korea; 9 Department of Animal Science and Technology, College of Biotechnology and Natural Resources, Chung-Ang University, Ansung-si, 17546 Republic of Korea

Correction to: *Scientific Reports* 10.1038/s41598-019-53102-8, published online 13 November 2019

This Article contains an error. The data generated in this study was originally deposited at the National Agricultural Biotechnology Information Center (NABIC) website, but is no longer available there. The Authors have deposited the data at NCBI, and therefore the corrected Data Availability section should appear as below:

## Data Availability

All the data generated in this study are freely available for download at NCBI. The accession number for whole genome data is PRJNA664646, and for RNAseq data is PRJNA662217.

