# Supplementary material for: Comprehensive genome and transcriptome analyses reveal genetic relationship, selection signature, and transcriptome landscape of small-sized Korean native Jeju horse
Source: Sci Rep. 2019 Nov 13;9:16672. doi: 10.1038/s41598-019-53102-8 (PMC6853925; doi:10.1038/s41598-019-53102-8)

Comprehensive genome and transcriptome analyses reveal genetic relationship, selection signature, and transcriptome landscape of small-sized Korean native Jeju horse

Krishnamoorthy Srikanth[1]†, Nam-Young Kim[2]†, WonCheoul Park[1], Jae-Min Kim[3], Kwon-Do Kim[4], Kyung-Tai Lee[5], Ju-Hwan Son[1], Han-Ha Chai[1], Jung-Woo Choi[6], Gul-Won Jang[1], Heebal Kim[4], Youn-Chul Ryu[7], Jin-Wu Nam[8], Jong-Eun Park[1], Jun-Mo Kim[9*], and Dajeong Lim[1]

Supplemental figure S1: Representative image of Jeju Horse

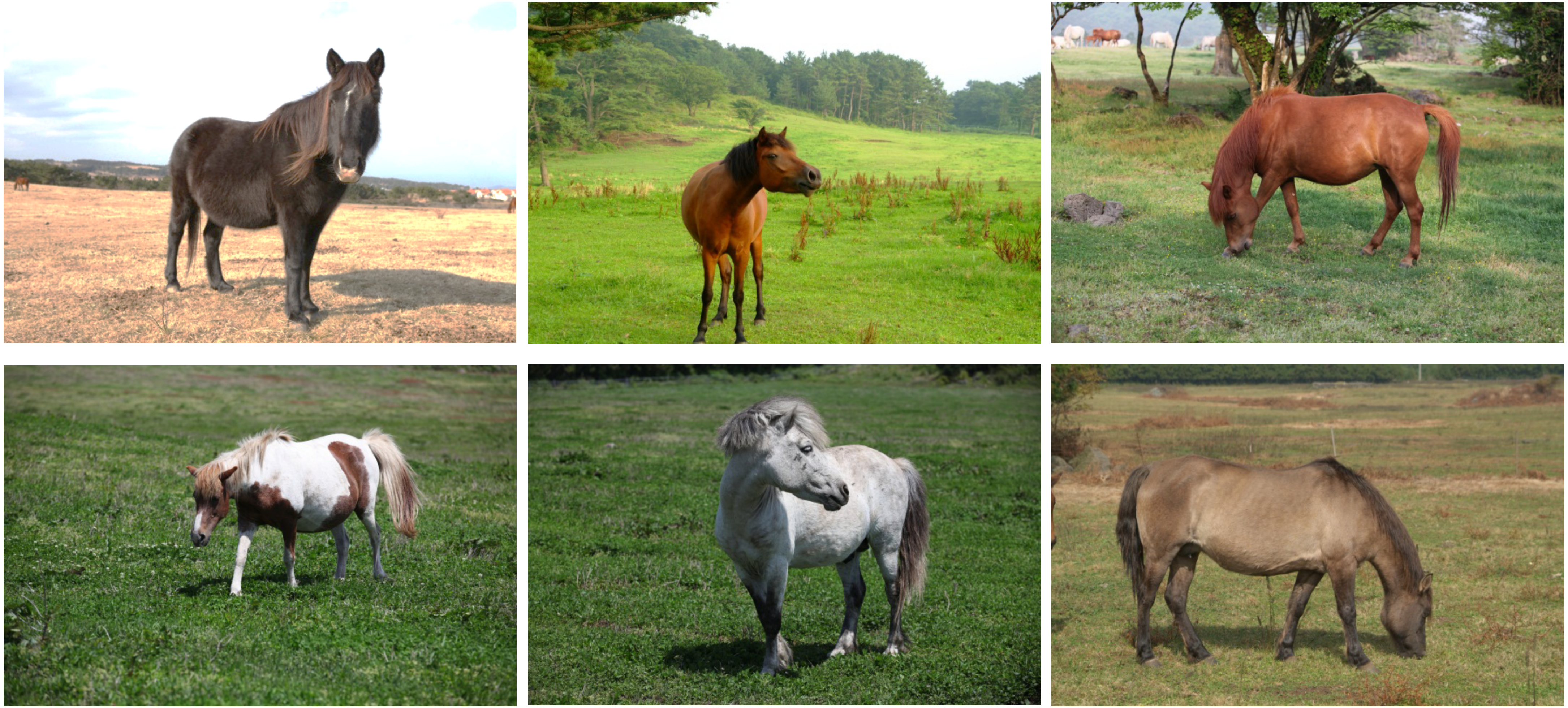

Supplemental figure S2: Pattern of population splits and gene flow between the breeds inferred from 0, 1 and 2 migration events (a, b and c respectively), d – f) are the corresponding covariance matrix

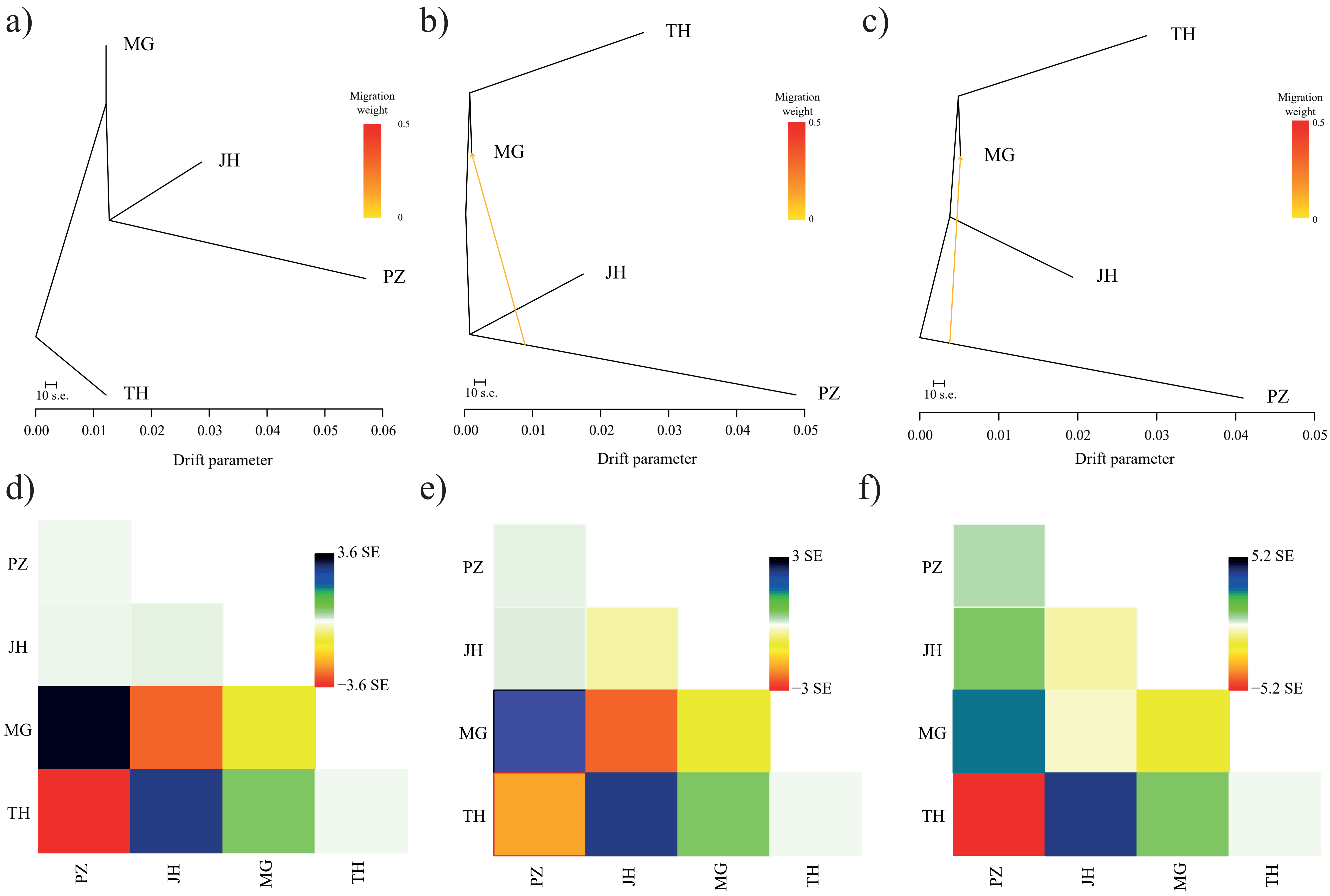

Supplemental figure S3: Percentage of Type1 fibers detected in the rump and thigh muscle of JH and TB.

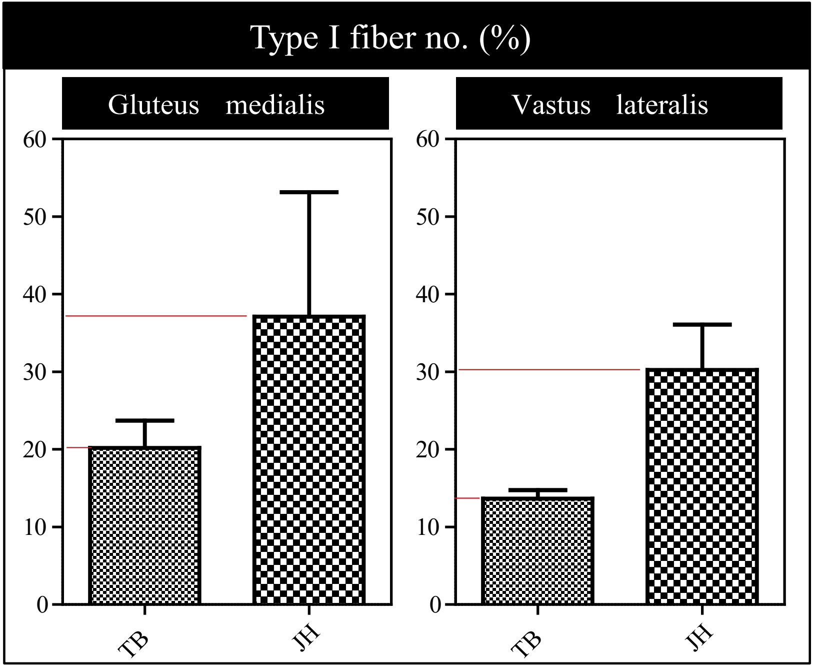

Supplement: Supplementary file 1 — Supplementary Figures [file 41598_2019_53102_MOESM1_ESM.pdf]
